# Physician Variability in Management of Emergency Department Patients with Chest Pain

**DOI:** 10.5811/westjem.2017.2.32747

**Published:** 2017-04-17

**Authors:** Peter B. Smulowitz, Orit Barrett, Matthew M. Hall, Shamai A. Grossman, Edward A. Ullman, Victor Novack

**Affiliations:** * Beth Israel Deaconess Medical Center, Harvard Medical School, Department of Emergency Medicine, Boston, Massachusetts; † Soroka University Medical Center and Faculty of Health Sciences, Ben-Gurion University of the Negev, Department of Medicine and Clinical Research Center, Be’er Sheva, Israel; ‡ Soroka University Medical Center and Faculty of Health Sciences, Ben-Gurion University of the Negev, Department of Internal Medicine, Be’er Sheva, Israel

## Abstract

**Introduction:**

Chest pain is a common emergency department (ED) presentation accounting for 8–10 million visits per year in the United States. Physician-level factors such as risk tolerance are predictive of admission rates. The recent advent of accelerated diagnostic pathways and ED observation units may have an impact in reducing variation in admission rates on the individual physician level.

**Methods:**

We conducted a single-institution retrospective observational study of ED patients with a diagnosis of chest pain as determined by diagnostic code from our hospital administrative database. We included ED visits from 2012 and 2013. Patients with an elevated troponin or an electrocardiogram (ECG) demonstrating an ST elevation myocardial infarction were excluded. Patients were divided into two groups: “admission” (this included observation and inpatients) and “discharged.” We stratified physicians by age, gender, residency location, and years since medical school. We controlled for patient- and hospital-related factors including age, gender, race, insurance status, daily ED volume, and lab values.

**Results:**

Of 4,577 patients with documented dispositions, 3,252 (70.9%) were either admitted to the hospital or into observation (in an ED observation unit or in the hospital), while 1,333 (29.1%) were discharged. Median number of patients per physician was 132 (interquartile range 89–172). Average admission rate was 73.7±9.5% ranging from 54% to 96%. Of the 3,252 admissions, 2,638 (81.1%) were to observation. There was significant variation in the admission rate at the individual physician level with adjusted odds ratio ranging from 0.42 to 5.8 as compared to the average admission. Among physicians’ characteristics, years elapsed since finishing medical school demonstrated a trend towards association with a higher admission probability.

**Conclusion:**

There is substantial variation among physicians in the management of patients presenting with chest pain, with physician experience playing a role.

## INTRODUCTION

Within the emergency department (ED) variation exists in the rate of testing and admission for a variety of clinical conditions. 1–5 This is likely multifactorial and linked to patient, ED, hospital, geographic, and physician-related factors. 6–15 While the emergency physician (EP) typically decides patient disposition, he or she may be influenced by a variety of issues beyond the patient’s clinical presentation. Fear of malpractice and risk aversion have both been demonstrated to be predictive of ED admission rates and testing for clinical conditions including chest pain and abdominal pain.^12^,^14^ In general, variation in care is a well-established marker of low-value care. For example, data from the Dartmouth Atlas in the United States (U.S.) and the National Health Service in the United Kingdom demonstrate that much of the variation is supply sensitive (related to the capacity or supply of the local healthcare system), and that “much of the variation in use of healthcare is accounted for by the willingness and ability of doctors to offer treatment rather than differences in illness or patient preference.” 16

Chest pain accounts for 8–10 million visits per year across the U.S., and about half of these patients are admitted to either an observation unit or inpatient service at a cost of $10–13 billion every year. 17 Evidence continues to accumulate that many of these are “low risk” chest pain patients who are unlikely to benefit from prolonged observation or additional cardiac risk stratification (e.g., stress testing or coronary computerized tomography [CT]). 18–22 Recently developed accelerated diagnostic pathways (ADPs) for chest pain, including the HEART score, have been demonstrated to reduce overall admissions for chest pain without exposing patients to major adverse cardiac events (MACE). 23–25 Growing data suggests that when low-risk criteria are met, additional testing offers no benefit and may increase the mortality rate for this particular subset of chest pain patients.^18^–^22^,^26^ In addition, modern-generation troponins (generally with 99th percentile of the upper reference limit in a healthy population of < 0.01 μg/L, even without considering high-sensitivity troponins not yet in use in the U.S.) can reliably exclude acute coronary syndrome (ACS)when done in serial testing without additional risk stratification. 27–29 On the other hand, one recent study of Medicare patients found an association between more conservative practice (higher admission rates) and lower incidence of acute myocardial infarction (MI) and death for this patient population. 30

We live in an era in which available pathways exist to risk stratify patients with chest pain and rapidly rule out ACS with increasing accuracy but where clinical guidelines suggest 72-hour provocative testing 31 and substantial medico-legal risk still pervades practice. Our major objective was to determine if chest-pain admission variation exists between physicians and what physician-related factors might predict this variation after controlling for appropriate patient and hospital factors. In essence, this paper purports to evaluate the extent to which variation exists in a common condition, and to elucidate some of the reasons why it might exist.

## METHODS

This was a single-institution retrospective observational study at a tertiary-care academic facility with an annual ED volume of approximately 55,000 patients. We included all ED visits from 2012 and 2013 with chest pain as the coded discharge ED or hospital diagnosis (*International Classification of Disease-9* codes 786.50, 786.51, 786.52, 786.59, and 413.9). Diagnoses for chest pain were obtained from the hospital administrative database, in which the ED diagnosis for discharged or ED observation patients and the inpatient diagnosis for admitted patients are recorded. We did not evaluate other surrogate markers of potential ACS like dyspnea, dizziness, and epigastric pain. We excluded patients with an elevated troponin (Troponin T ≥0.01 ng/ml) or an ECG demonstrating an ST elevation myocardial infarction since there is unlikely to be any variation around admission rates for patients with obvious ACS. ED visit and admission-level information were obtained from administrative hospital databases. We included physicians with a minimum of 30 patient encounters for chest pain and stratified physicians by age, gender, years since finishing medical school, and residency location (our institution versus other institutions). Since most of this study predates more recent literature on accelerated diagnostic pathways like the HEART score and the 2015 data on the short-term safety of patients with normal ECGs and two normal troponins, decisions in this study were made by individual discretion and not based on a particular accelerated diagnostic pathway.^23^,^24^,^29^

**Population Health Research Capsule**

What do we already know about this issue?

There is substantial variation in rates of admission from the ED for patients with chest pain.

What was the research question?

Are there factors related to the individual physician that predict this variation?

What was the major finding of the study?

After controlling for patient level variables, physician-level factors are associated with variation in admission rates.

How does this improve population health?

Interventions directed at physician decision-making may reduce admission

rates and potentially unnecessary cardiac testing, procedures, and costs.

We divided the study population into “discharge” and “admission” groups. For the purposes of this study we considered patients placed in observation status (either in the ED or medical floor) to be part of the admission arm. Consistent with current literature and Medicare billing rules, we included any patient with an observation order and two sets of cardiac markers, but a length of stay (LOS) under eight hours, in the “discharge” group. All patients with an observation order and LOS over eight hours were included in our admission group. 30 In our administrative databases we were unable to distinguish between patients placed in observation status in the ED versus those placed in observation status on the inpatient floor. Patients kept in our ED observation unit typically have a LOS over eight hours and under 24 hours, and typically have stress testing performed prior to discharge. Those discharged in under eight hours are still likely to have at least one (more often two) cardiac biomarkers drawn, but then are discharged without additional testing based on a classification as “low risk” chest pain. Thus, the key clinical distinction is whether a patient was felt to be low enough risk to be discharged without prolonged observation or additional provocative testing. This distinction is our anticipated root cause of variance in practice patterns among physicians, which was our primary outcome of interest.

The study was approved by the institutional review board at our institution.

### Primary Data Analysis

We used patient-visit as the unit for the univariate analysis and multivariate models, adjusted for the repeated visits. Patient-visit characteristics are presented as mean ± standard deviation (SD) for continuous variables and as percentage for categorical variables. Categorical variables were compared using the chi-square test. We examined continuous variables using unpaired T-testing. Non-parametric variables were compared with Mann-Whitney test.

We assessed individual physicians’ rates of admissions by a multivariable logistic regression model using generalized estimation equation (GEE) method, which accounted for clusters of multiple visits by the same patient. Covariance matrix was conservatively defined as unstructured. Variable selection in multivariable modeling was based on clinical and statistical significance. We included the following patient-level variables into the models: patient age, previous ischemic heart disease, hypertension, dyslipidemia, diabetes mellitus, glucose (in increments of 10 mg/dL) and creatinine levels. Physician characteristics were also included in the model and included gender, residency location, and years since medical school graduation. We reported final parsimonious models.

A two-sided P value <0.05 was considered statistically significant. We performed all statistical analyses using SPSS 22.0 (SPSS Inc. Chicago, Illinois, USA).

## RESULTS

### Study Size

**Figure 1:**
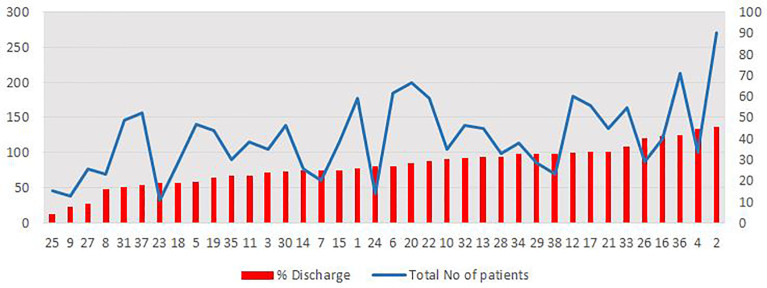
Summary of total patients with chest pain (left vertical axis) who were seen and percent discharged (right vertical axis) by physician. Each red bar and blue dot pair represents an individual physician (n=38).

Of 4,585 total patient visits (3,917 distinct patients) presenting with chest pain in the two-year period, 4,577 had documented dispositions. Of these, 3,252 (70.9%) were either admitted to the hospital or into observation status, while 1,333 (29.1%) were discharged after evaluation in the ED. Median number of ED visits per physician was 132 (IQ range 89–172). Average admission rate per physician was 73.7±9.5% ranging from 54% to 96% (Figure 1 presented as rate of discharges). A sizeable majority of the admissions (2,638/3,252; 81.1%) were to observation.

### Characteristics of Study Subjects

Mean age in the discharged group was 44 years (±17.3) and 59 years (±14.3) in the admission group (p<0.001). There was statistically significant variation between the prevalence of clinical risk factors and comorbidities including coronary artery disease (CAD), hypertension, diabetes, dyslipidemia (p < 0.001) and atrial fibrillation/flutter (p = 0.032) between the groups. Patient demographic and clinical characteristics for discharge versus admission groups are noted in Table 1. Physician level characteristics are demonstrated in Table 2.

### Admission Risk

Results of the unadjusted analysis are displayed in Table 3. In terms of unadjusted factors at the patient level, female patients were less likely to be hospitalized compared to male patients (odds ratio [OR]=0.773; p<0.001 95% confidence interval [CI] [0.680–0.879]). Black patients were less likely to be hospitalized compared to white patients (OR 0.736, p<0.001, 95% CI [0.40–0.846]). Older patients had a higher likelihood of admission (OR=1.066, p<0.001; 95% CI [1.061–1.072]). Comorbidities associated with a higher likelihood of admission included diabetes mellitus (OR=2.199; p<0.001; 95% CI [1.724–2.806]), hypertension (OR=2.203; p<0.001; 95% CI [1.724–2.806]), CAD (OR=3.034; p<0.001 95% CI [2.164–4.252]), dyslipidemia (OR=1.889; p<0.001; 95% CI [1.483–2.407]), and prior cardiac dysrhythmias (OR=1.778; p=0.034; 95% CI [1.045–3.025]). Higher initial glucose and creatinine levels were also significantly associated with higher admission rates (OR=1.007; p<0.001; 95% CI [1.005–1.009] and OR=1.558; p<0.001; 95% CI [1.216–1.998], respectively).

With respect to physician-related factors, female physicians were 1.4 times more likely to admit compared to male physicians (OR=1.415; p<0.001; 95% CI [1.214–1.648]). Physicians with greater patient volume were less likely to admit. (OR=0.995; p<0.001; 95% CI [0.995–0.997]). In the univariate analysis, neither residency location nor duration of experience (p = 0.24) were predictive of admission risk.

**Figure 2:**
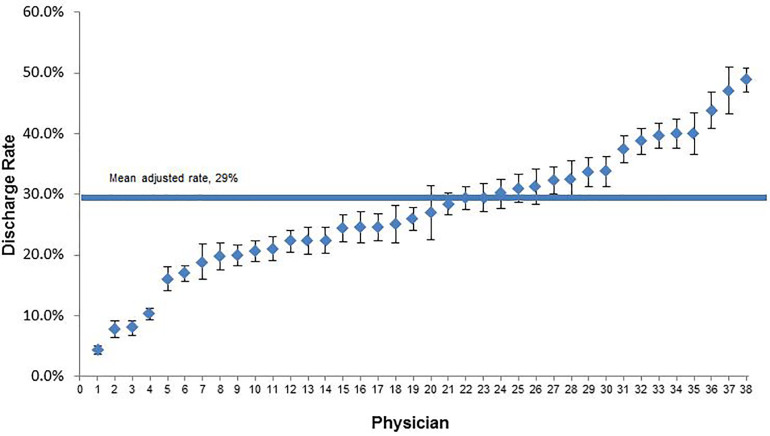
Adjusted physician-level variation in discharge rates represented by likelihood of discharge compared to average discharge rate.

After controlling for the potential confounders, significant variation remained in admission rate at the individual physician level with adjusted OR ranging from 0.42 to 5.8 as compared to the average admission rate. Factors found to be significant in a multivariate analysis of patient- and physician-level factors include male patient gender, patient age, hypertension, and history of coronary artery disease, with greater physician experience demonstrating a trend towards significance (OR 1.85, p 0.095, 95% CI [0.09 – 3.81]) (Table 4). We assessed the model performance by analyzing c-statistics. C-statistics for the model was 0.78 (95% CI [0.74–0.86]). After controlling for the potential confounders, significant variation remained in admission rate at the individual physician level with adjusted OR ranging from 0.42 to 5.8 as compared to the average admission rate (Figure 2).

## DISCUSSION

Our data supports the initial hypothesis that variation exists in admission and rates for patients presenting to our hospital with chest pain, and suggests that this variation is at least to some degree attributable to physician-related factors. This variation persists despite major improvements in the sensitivity of troponins to adequately rule out potential ACS acute coronary syndromes. 26, 27

Of note, while there are multiple factors within the univariate analysis that suggest factors with significant correlation to admission, only a few of these factors remain relevant when controlling for potential confounders. Additionally, it is not surprising that a few of the patient-level factors (namely age, comorbidities, and abnormal lab results) are associated with admission. What is interesting – and the main finding of this paper – is that after controlling for potential confounders, considerable variation in rates of admission exists that is at least to some degree attributable to physician-level factors.

Within the domain of chest pain, while it is possible that some of this variation will be eliminated by the adoption of new ADPs, this study simply affirms the presence of a substantial amount of variation at the physician level in one of the most common clinical conditions presenting to EDs worldwide. One of the most surprising features was the actual breadth of variation between physicians practicing at the same facility. This underscores the importance of variation as a general phenomenon in healthcare, both likely in terms of intensity of testing and selection of patient disposition, as well as the central role of the physician as the main driver of variation. In general, variation is understood to be a marker of low-value care. Variation is prevalent across many conditions both within emergency care and other areas of healthcare, and it has been previously demonstrated to be related to several domains including patient, ED, hospital, geographic, and physician-related factors. 6–15,32–35 Thus, while our paper focuses on chest pain, we suspect these findings will be generalizable to other conditions within emergency care.

One of the interesting features of our findings was the trend towards an association of greater physician experience with greater rates of admission. There are many potential explanations for this. Older physicians may lag in terms of education with respect to the increased sensitivity of newer generation cardiac biomarkers, may be simply less likely to modify practice patterns to novel techniques, or may be overall less tolerant of risk. It is possible that with greater experience comes a greater appreciation for unanticipated outcomes, or that physicians are simply more likely to experience a lawsuit the longer they practice. Previous studies have been mixed in terms of the impact of physician experience on levels of testing and admission.^10^,^36^,^37^ Further work will be necessary to clarify the exact impact and role of experience, how it differs for different clinical conditions, and how it interacts with risk tolerance.

Whatever the cause of physician-related variation in chest pain admission, this phenomenon suggests that interventions at the level of the physician – including evidence-based pathways and modern ADPs – may have the potential to provide support for decision-making and reduce variation in practice patterns and in turn reduce healthcare costs. Our results suggest that establishment of an ADP in our institution may help reduce variation and over-reliance upon observation or hospital admissions by establishing an evidence-based approach to risk stratification. However, even the HEART score relies on clinician gestalt as one of its major decision points, which may limit its effectiveness in reducing existing variation and admission rates. 24

## LIMITATIONS

Our most notable limitation is that this is a single-institution study. While this may limit the generalizability of the results, we believe the findings are consistent with existing literature with respect to variation in practice patterns. Another potential limitation is that we did not discern between ED observation status and inpatient observation status. While our cutoff of eight hours was intended to include in the “admission” group only those that were intended to receive prolonged evaluation, this still may not accurately reflect the thought process of the ordering physician. It is clear that this designation of “observation” patients as “admissions” may overestimate our overall percent of patients classified as admitted. While our true rate of admissions is undoubtedly lower than the roughly 70% found in our study, this does not reduce the impact of the observed variation in admission rates. Our true admission rate is likely higher than average, perhaps driven in part by a relatively conservative practice style in the Northeast U.S.. Furthermore, while in many hospitals in the U.S. patients kept in ED observation units might be counted as discharges, we consider these more appropriate to be counted as admissions since the majority of these patients at our institution will have serial cardiac biomarkers and provocative cardiac testing and therefore accomplish the same evaluation as commonly occurs during an inpatient admission, while those in observation status for less than eight hours typically only undergo two troponin tests without more advanced testing.

Once adjusted for observation stays, our rates would not be unusual for the U.S. The study by Cotterill et al. of Medicare patients found a wide swing in admission rates, with an average rate of 63% and the lowest and highest quintiles ranging from 38 to 81%. 30 We anticipate these numbers will drop with the implementation of modern ADPs.

A further limitation is our lack of data on basic measures of major adverse cardiac events (acute MI, positive cardiac catheterization, cardiac bypass surgery) and the lack of follow-up on outcomes. This was largely intentional since we were aiming to evaluate variation as an outcome and not the safety of decision-making.

We also did not include potential surrogate symptoms of ACS like dyspnea, dizziness, and epigastric pain. The goal of this study was to evaluate whether variation existed for the work-up of chest pain, not all potential presentations of ACS. We anticipate even greater variability in how physicians risk-stratify these other types of commonly presenting symptoms.

Additionally, this was a retrospective study using a hospital dataset that is subject to the limitations inherent in retrospective investigations.

## CONCLUSION

In our single-institution study there is substantial existing variation at the physician level in the management of patients presenting with chest pain with a trend towards higher admission rates correlated with greater physician experience. It would be important to know the interaction between physician experience level and risk aversion. Additionally, novel ADPs may moderate the variation in and absolute rate of testing and admission for patients presenting with low-risk chest pain.

## Figures and Tables

**Table 1:** Patient demographic and clinical characteristics in study of variation in rates of admission from the ED for patients with chest pain.

Variable	Discharge, n = 1333	Admission, n = 3252	p value
Age, mean (SD)	44 (17.3)	59 (14.3)	< 0.001
Gender, n male (%)	570 (42.8)	1598 (49.1)	< 0.001
Race, n (%)			
White	847 (63.8)	2265 (70)	< 0.001
Black	436 (32.8)	858 (26.5)	< 0.001
Asian	40 (3.0)	86 (2.7)	< 0.001
Co-morbidities, n (%)			
Coronary artery disease	40 (3.0)	279 (8.6)	< 0.001
Diabetes mellitus	84 (6.3)	419 (12.9)	< 0.001
Congestive heart failure	2 (0.2)	12 (0.4)	0.376
Hypertension	243 (18.2)	1071 (32.9)	< 0.001
Dyslipidemia	87 (6.5)	379 (11.7)	< 0.001
Smoking	34 (2.6)	84 (2.6)	0.950
Atrial fibrillation/flutter	17 (1.3)	73 (2.2)	0.032
CVA/TIA	4 (0.3)	9 (0.3)	1

*CVA*, cerebrovascular accident; *TIA,* transient ischemic attack.

**Table 2:** Individual physician characteristics.

Variable	Physicians (n, %), total = 38
Gender	
Male	28 (73.7)
Medical school in the USA	35 (92.1)
Residency location	
Study hospital	12 (31.6)
Other hospital	26 (68.4)
Years since medical school, n (%)	
<5	4 (10.5)
6–10	10 (26.3)
11–20	17 (44.7)
>20	7 (18.4)

**Table 3:** Univariate analysis of patient- and physician-level characteristics’ impact on variation in admission rates of patients with chest pain.

Variable	Odds ratio	95% CI, lower limit	95% CI, upper limit	p value
Patient age	1.066	1.061	1.072	<0.001
Patient gender	0.773	0.680	0.879	<0.001
Race (Reference white)				
Black	0.736	0.640	0.846	<0.001
Asian	0.804	0.548	1.180	0.804
Patient comorbidity				
Smoking	1.013	0.677	1.517	0.950
Dyslipidemia	1.889	1.483	2.407	<0.001
Diabetes mellitus	2.199	1.724	2.806	<0.001
Hypertension	2.203	1.724	2.806	<0.001
Coronary artery disease	3.034	2.164	4.252	<0.001
Congestive heart failure	1.465	0.551	11.128	0.238
Cardiac arrhythmia	1.778	1.045	3.025	0.034
Stroke/transient ischemic attack	0.922	0.283	2.999	0.893
Creatinine	1.558	1.216	1.998	<0.001
Glucose	1.007	1.005	1.009	<0.001
Troponin	4.066	0.304	54.420	0.289
Number patients per physician	0.996	0.995	0.997	<0.001
Years since medical school	0.995	0.986	1.004	0.244
Residency within study institution	0.983	0.862	1.122	0.804
Attending gender	1.415	1.214	1.648	<0.001

**Table 4:** Multivariate analysis of patient- and physician-level characteristics’ impact on variation in admission rates of patients with chest pain.

	Odds ratio	95% CI, lower limit	95% CI, upper limit	p value
Male patient gender	1.34	1.17	1.54	<0.001
Age above 60 years	3.35	2.85	3.95	<0.001
Hypertension	1.42	1.21	1.68	<0.001
Diabetes mellitus	1.74	1.33	2.27	<0.001
History of CAD	2.28	1.58	3.30	<0.001
5 or more years from medical school graduation	1.85	0.90	3.81	0.095
Individual physician^*^				<0.001

*CAD*, coronary artery disease.

Adjusted odds ratio shown in Figure 2.
